# Combined fractional anisotropy and subcortical volumetric deficits in patients with mild-to-moderate depression: Evidence from the treatment of antidepressant traditional Chinese medicine

**DOI:** 10.3389/fnins.2022.959960

**Published:** 2022-08-23

**Authors:** Yuan Li, Junjie Wang, Xu Yan, Hong Li

**Affiliations:** ^1^Department of Radiology, Heping Hospital Affiliated to Changzhi Medical College, Changzhi, China; ^2^Shanxi Key Laboratory of Artificial Intelligence Assisted Diagnosis and Treatment for Mental Disorder, First Hospital of Shanxi Medical University, Taiyuan, China; ^3^Department of Psychiatry, First Hospital/First Clinical Medical College of Shanxi Medical University, Taiyuan, China; ^4^Department of Medical Imaging, Changzhi Medical College, Changzhi, China; ^5^Department of Mental Health, Shanxi Medical University, Taiyuan, China

**Keywords:** mild-to-moderate depression, fractional anisotropy, subcortical volume, dorsolateral prefrontal cortex, Shuganjieyu capsule

## Abstract

Numerous neuroimaging studies have demonstrated that diverse brain structural plasticity could occur in a human brain during a depressive episode. However, there is a lack of knowledge regarding the underlying mechanisms of mild-to-moderate depression (MMD), especially the changes of brain structural characteristics after treatment with the Shuganjieyu capsule (SG), a kind of traditional Chinese medicine that has been recommended for the specialized treatment of MMD. In this study, we investigated the structural brain plasticity in MMD that have been undergoing 8 weeks of SG treatment compared with age- and sex-matched healthy controls (HCs) and assessed the relationship between these brain structural alternations and clinical symptoms in MMD. At the baseline, we found that: (1) fractional anisotropy (FA) values in patients with MMD were found to be significantly increased in the regions of anterior limb of internal capsule (ALIC) [MNI coordinates: Peak (*x*/*y*/*z*) = 102, 126, 77; MMD FA_*peak*_ (Mean ± SD) = 0.621 ± 0.043; HCs FA_*peak*_ (Mean ± SD) = 0.524 ± 0.052; MMD > HCs, *t* = 9.625, *p* < 0.001] and posterior limb of internal capsule (PLIC) [MNI coordinates: Peak (*x*/*y*/*z*) = 109, 117, 87; MMD FA_*peak*_ (Mean ± SD) = 0.694 ± 0.042; HCs FA_*peak*_ (Mean ± SD) = 0.581 ± 0.041; MMD > HCs, *t* = 12.90, *p* < 0.001], and FA values were significantly positively correlated with HAMD scores in patients with MMD. (2) Patients with MMD showed smaller gray matter volume (GMV) of the dorsolateral prefrontal cortex (DLPFC), frontal cortex, occipital cortex, and precuneus, and the GMV of DLPFC was negatively correlated with HAMD scores. After SG treatment, we found that (1) the HAMD scores decreased; (2) FA values were significantly decreased in the regions of the ALIC and PLIC compared to those at baseline and TBSS revealed no significant differences in FA values between patients with MMD and HCs. (3) The structural characteristics of DLPFC in patients with MMD obtained at the 8th week were improved, e.g., no significant differences in GMV of DLPFC between the two groups. Taken together, our results provided neuroimaging evidence suggesting that SG is an effective treatment for patients with MMD. Moreover, alterations of GMV after 8 weeks of SG treatment indicated a potential modulation mechanism in brain structural plasticity within the DLPFC in patients with MMD.

## Introduction

Depression is a notably prevalent mental disorder, characterized by depressed mood, loss of interest, and increased fatigue, affecting an estimated 264 million people worldwide (Disease et al., 2018). Globally, the prevalence of depression is ~4.4%, and it has become the main factor contributing to the global disease burden. There are more than 95 million depressive patients in China, with a lifetime prevalence of 6.9% and a 12-month prevalence of 3.6% (Huang et al., 2019). Depending on the severity of the symptoms, depression can be categorized as mild, moderate, or severe. Mild-to-moderate depression (MMD) involves fewer than five symptoms of depression and is more common than severe depression in general medical settings (Liu et al., 2020; Meng et al., 2021).

With the development of neuroimaging technology, a growing number of researchers have begun to use magnetic resonance imaging (MRI) to study the structural and functional features of the brain in patients with depression. Structural imaging techniques are useful tools for detecting neurobiological alterations associated with depression. Surface-based morphometry based on anatomical T1-weighted MRI provides better alignment of cortical landmarks over the whole brain using vertex-by-vertex analysis to explore possible changes in the gray matter (GM) (Mills and Tamnes, 2014). Tract-based spatial statistics (TBSS) based on diffusion tensor imaging (DTI) implements voxel-wise statistical analysis of fractional anisotropy (FA) data to examine changes in the microstructure of white matter (WM) bundle fibers (Zhang H. et al., 2012). Structural neuroimaging studies have shown that various structural abnormalities can occur in the human brain during depressive episodes. Researchers have shown that depression is associated with general volume reductions in several brain regions, such as the frontal cortex, mainly the prefrontal cortex (PFC), orbitofrontal cortex (OFC), amygdala, thalamus, insula, and hippocampus (Kronenberg et al., 2009; Ancelin et al., 2019). Koolschijn et al. found that major depressive disorder (MDD) is characterized by a reduction in volume in several frontal regions, including the OFC and anterior cingulate cortex (ACC), which is attributed to dysfunctions in stress regulation and emotional processing (Koolschijn et al., 2009). In addition, the number of MDD was positively correlated with the degree of hippocampal volume reduction, suggesting that repeated depressive episodes may lead to a further reduction in hippocampal volume (Eker and Gonul, 2010). In another study, Bremner et al. found that compared with healthy controls (HCs), the left hippocampal volumes of patients with MDD were smaller (Bremner et al., 2002). Furthermore, other researchers found that the volumes of other brain regions, such as the frontal lobe, caudate, amygdala, and temporal lobe, were not significantly different between patients with MDD and HCs (Koolschijn et al., 2009).

For the visualization of brain networks, WM tract imaging technology has been widely used and FA is the most common variable used in DTI studies (Catani, 2006). Furthermore, FA abnormalities in the fiber tracts of individuals with MDD may indicate microstructural changes underlying the pathophysiology of the disorder. For example, Liao et al. performed a meta-analysis to identify areas of MDD WM change. They found that FA values were reduced in the WM tracts connecting the PFC to different brain cortices (temporal, occipital, and frontal lobes) and subcortical regions (amygdala and hippocampus), demonstrating the presence of structural network alterations in MDD (Liao et al., 2013). Furthermore, these researchers have used DTI technology to reveal that patients characterized with impaired WM integrity of fiber tracts that contribute to emotional regulation, including the superior longitudinal fasciculus, uncinate fasciculus, corpus callosum, anterior corona radiata, cingulum, and inferior fronto-occipital fasciculus (Kieseppa et al., 2010; Cole et al., 2012; Jiang et al., 2017; Vilgis et al., 2017).

Antidepressant medication is a first-line treatment for severe MDD (Cleare et al., 2015), and has been shown to ameliorate functional impairment, with changes in neural activation and brain structure (Dichter et al., 2015; Klauser et al., 2015). In recent years, imaging studies on the effects of different mechanism drugs on brain structure in patients with MDD have increased rapidly (Zeng et al., 2012; Groman et al., 2013; Yuen et al., 2014; Dusi et al., 2015; Fu et al., 2015; Liu et al., 2017). These studies have proved that whether the drug mechanism is the same or not, antidepressants can improve depression by changing brain structure, and the changes in the volume of different brain regions are closely related to the neuroplasticity of antidepressants. On the other hand, the brain areas improved by antidepressants with different mechanisms may also be different, which may be closely related to the different distribution sites of the brain-related neurotransmitters, and also have a great relationship with the occupancy of their neurotransmitter serum transporters.

Although previous studies have investigated structural abnormalities in patients with MDD, the underlying mechanisms of MMD, especially the changes in brain structural characteristics after drug treatment, remain unclear. Furthermore, MMD is the initial stage of a depressive disorder and is more common than severe depression in the general medical environment. Exploring the structural features of MMD provides a basis for the mechanism of MDD pathogenesis and may lead to optimized treatment for patients in clinical practice.

Shugan Jieyu Capsule (SG) is a Chinese herbal medicine mainly composed of *Eleutherococcus senticosus* (also known as *Acanthopanax senticosus*) and *Hypericum perforatum* (also known as St. John's wort) (~5:6). Both *E. senticosus* and *H. perforatum* have been proven to be effective in the treatment of depression and impaired cognition (Sithisarn et al., 2013; Ben-Eliezer and Yechiam, 2016). Furthermore, SG has been licensed and widely prescribed for the treatment of depression in China since 2008. The effect of SG in ameliorating depressive symptoms is similar to that of escitalopram, but owing to its fewer adverse reactions in relieving the symptoms of depression, it has been recommended for the specialized treatment of MMD (Zhang et al., 2014).

Previous studies have revealed that GM and WM in certain brain regions of patients with MDD are abnormal, and these brain regions are usually involved in individual emotion processing. The initial stage of depressive disorder is MMD, and it mainly presents in the form of emotional problems, which is a common clinical symptom. Therefore, we hypothesized that modified volumes and integrity of the GM and WM in certain brain regions could occur in patients with MMD, and these changes may be associated with clinical variables. Furthermore, depressive symptoms could be reduced after SG treatment, and the efficacy of SG in MMD might be reflected in the associated changes in the structural differences in brain regions. To test our hypothesis, we combined psychometrics and structural MRI techniques to investigate brain structural alterations in 47 patients with MMD following SG treatment compared with 43 age- and sex-matched HCs.

## Methods and materials

### Participants

Forty-seven right-handed patients with MMD (17 men; mean age 26.12 ± 5.06 years) and 43 age- and sex-matched HCs (13 men; mean age 26.27 ± 5.69 years) participated in the study. All patients satisfied the following inclusion criteria: (1) Diagnostic and Statistical Manual of Mental Disorders-Fourth Edition (DSM-IV) criteria for MDD diagnosis with an actual mild-to-moderate episode; (2) score 7 < HAMD-24 scores <24; (3) 18 years ≤ age ≤ 50 years; (4) right-handed (Edinburgh Handedness Inventory, EHI) (Oldfield, 1971); (5) no antidepressant treatment in the past 2 months; and (6) no comorbid Axis-I diagnosis, which comprises all psychological diagnostic categories except intellectual disability and personality disorder. It is worth noting that older patients have an increased risk of vascular disease, which may affect brain structure. To mitigate the influence of vascular factors on brain structure (Kendler et al., 2009), the participants in this study were adults under the age of 50. In fact, considering the vascular factors, we actively encouraged younger patients to participate in this study. Exclusion criteria included a history of neurological diseases, past or current history of alcoholism, elevated risk of suicide, and contraindications of MRI scans (e.g., implanted metal objects and claustrophobia). Forty-three age- and sex-matched, right-handed HCs were recruited from the community nearby and were accessed *via* the Structured Clinical Interview for DSM-IV, non-patient edition (SCID-I/NP) to exclude the diagnosis of MMD, as well as other axis-I psychiatric disorders. In addition to the above, the other criteria also included: (1) HAMD-24 scores <7 and (2) no family history of mental illness. (3) There were no psychological or psychopathological symptoms. The exclusion criteria for the HCs were identical to those for MMD. The participants signed an informed consent form to participate in this study. This study was approved by the Ethics Committee of Shanxi Medical University.

### Clinical assessments

For patients with MMD, clinical assessments included age at illness onset and the severity of illness. Depressive symptoms and anxiety symptoms were independently assessed based on HAMD-24 and HAMA-14 by two research psychiatrists. The clinical assessment of HCs included the HAMD and HAMA. The HAMD was established in 1960 and adopts a 5-level rating scale from 0 to 4 points. The total score ranges from 0 to 78, and the depression level can be divided as follows: <8, no depression; 8–24, MMD; and > 24, severe depression. The HAMA was established in 1959 and adopts a 5-level rating scale from 0 to 4 points. The total score ranges from 0 to 56, and the anxiety levels can be divided as follows: <7, no anxiety; 7–14, possible anxiety; 15–21, anxiety; 21–29, obvious anxiety; and >29, severe anxiety. Both scales were confirmed to have good reliability.

### Medication treatment

After the pre-tests, patients included were instructed to take a Chinese herbal medicine named SG, which is mainly made up of *Acanthopanax* and *H. perforatum*, licensed and widely prescribed for depression in China since 2008. Patients with MMD received SG treatment for 8 weeks after the baseline MRI scans. According to the recommended medication dose, patients with MMD took 1.44 g of SG per day, including 0.72 g in the morning and 0.72 g in the evening for 8 weeks. During treatment, all participants were followed up weekly *via* telephone interviews to maintain good adherence. After treatment, all the participants completed the HAMD and HAMA assessments.

### MRI acquisition

MR images were acquired using a 3.0-T Siemens Skyra MRI scanner (Siemens Medical, Erlangen, Germany) at the Shanxi Provincial People's Hospital. A magnetization-prepared rapid gradient echo sequence was used to acquire high-resolution T1-weighted anatomical images (repetition time = 2,530 ms, echo time = 2.01 ms, inversion time = 900 ms, flip angle = 9°, resolution matrix = 256 × 256, slices = 192, thickness = 1 mm, voxel size = 1.0 × 1.0 × 1.0 mm^3^, and duration = 7 min). The diffusion data for each subject were obtained using a diffusion-weighted, single shot, spin-echo, echo planar imaging (EPI) sequence (TR/TE = 10,200/91 ms, matrix = 96 × 96, FOV = 192 × 192 mm, voxel size = 2.0 × 2.0 × 2.0 mm^3^, 70 axial slices, 2.0 mm slice thickness, *b*-value 1 = 0 s/mm^2^, and *b*-value 2 = 1,500 s/mm^2^) in 64 directions. Resting-state fMRI data were acquired using an EPI sequence (TR/TE, 2620/30 ms; flip angle: 90°, FOV 192 ×192 mm; matrix, 64 ×64; slice thickness, 3 mm; slices, 47; 220 volumes).

### Voxel-based morphology analysis

The GM volume was examined using VBM from the FSLVBM. All T1-weighted images were first extracted from the brain and then segmented into GM, WM, or cerebrospinal fluid. A GM template was generated by registering and averaging all GM images. The GM image for each participant was then registered to the template using a non-linear transformation. A voxel-wise permutation test was used to identify significant group differences between patients with MMD and HCs to a distribution generated from 5,000 permutations of the data for each voxel of the template, using a sigma filter of 3 mm for smoothing. *P* < 0.05 (FWE, using the TFCE method) was considered statistically significant.

### Tract-based spatial statistics

We analyzed WM properties using voxel-wise TBSS from the FMRIB Software Library (FSL) (https://fsl.fmrib.ox.ac.uk/fsl/fslwiki/TBSS) (Smith et al., 2006). This method identified a core WM “skeleton” that was anatomically equivalent across participants. First, diffusion data were corrected using Eddy correction (eddy_correct) and brain-extracted using BET in FSL (Smith et al., 2002). The FA images were created by fitting a tensor model using DTI fit, and then FA data from all participants were aligned into a common standard space using the non-linear registration tool, FNIRT. Next, the mean FA image was thinned to create a mean FA skeleton representing the centers of all tracts common to the group. The FA images from individual subjects were then projected onto this skeleton and the resulting data were fed into voxel-wise cross-subject statistics. The significant regions were thickened for visualization using the TBSS fill script in the FSL. The Johns Hopkins University ICBM-DTI-81 WM labels atlas was used to locate anatomical structures in the MNI152 spaces. Independent-sample *t-*tests were conducted to examine FA differences between the two groups using the non-parametric permutation tool *Randomize* in FSL.

### Statistical analysis

For the demographic data, group differences in age and education level were evaluated using independent sample *t*-tests, and sex differences were assessed using Pearson's chi-square test. The difference between HAMA and HAMD scores of patients before and after treatment was tested by paired sample *t*-test. Pearson's correlation analyses were performed to assess the relationship between structural deficits and depressive symptoms in the MDD group.

Furthermore, to assess the joint influence of structural deficits on depression, a multiple linear regression analysis based on a forward stepwise selection procedure was applied. In this analysis, depression symptom severity (overall HAMD scores) was used as the dependent variable, and structural features (GM volume and FA values) were used as explanatory variables. The forward stepwise selection procedure started by including the explanatory variable that could mostly and significantly explain the dependent variable (*p* < 0.05) and adjusted by repeatedly adding other explanatory variables (if any) that could significantly improve the fit of the model (*p* < 0.05), until none could improve the model. Before multiple linear regression analysis, all variables were checked for deviation from normality using the Kolmogorov-Smirnov test, and no significant deviation from normality was observed for any variable (*p* > 0.05).

## Results

### Demographic and clinical characteristics

**Figure 1:**
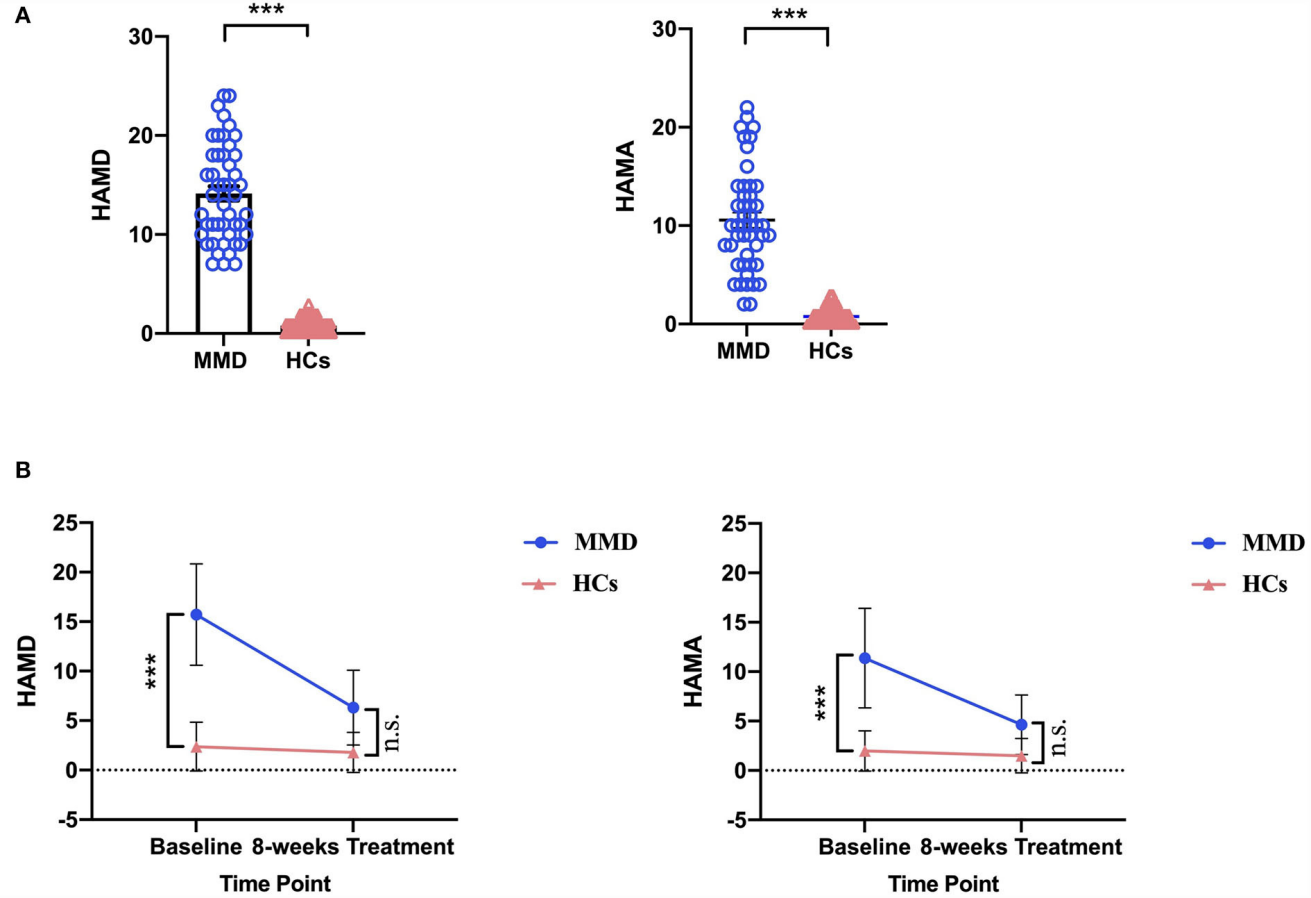
Comparison of HAMD and HAMA scores between MMD patients and HCs. **(A)** At the baseline, MMD patients exhibited significantly larger HAMD and HAMA scores than in HCs. The error bars indicate standard error. **(B)** After 8 weeks SG-treatment, the HAMD and HAMA scores of MMD patients decreased significantly, and there were no significant differences in HAMD and HAMA scores of MMD patients compared HCs. HAMD, hamilton depression; HAMA, hamilton anxiety; MMD, mild-to-moderate depression; HCs, healthy controls. ***, *p* < 0.001; n.s., no significance.

Demographic (i.e., age, sex, and years of education) and clinical variables in patients with MMD and HCs are summarized in Table 1. Although sex, age, and years of education were not significantly different between the two groups, the clinical variables, as quantified by 24-item Hamilton Depression Rating Scale (HAMD) (*t* = 16.060, *p* < 0.001; Table 1 and Figure 1A) and 14-item Hamilton Anxiety Rating Scale (HAMA) (*t* = 12.220, *p* < 0.001; Table 1 and Figure 1A), were significantly larger in patients with MMD than in HCs. Patients with MMD received SG treatment for 8 weeks, and the symptoms were significantly reduced from baseline to the end of SG treatment. Specifically, 37 patients had HAMD scores <7, and 10 patients had HAMD scores between 7 and 14. At the same time, 32 patients had HAMA scores <7, and 15 patients had HAMA scores between 7 and 14. In addition, the mean HAMD and HAMA scores were <7 in patients with MMD after 8 weeks of SG treatment (Table 2 and Figure 1B). Therefore, it can be considered that after 8 weeks of SG-treatment, the condition of all patients has been effectively alleviated.

**Table 1:** Participant demographics.

	**MMD (47)**	**HCs (43)**	**df**	**χ^2^/t value**	***p*-value**
	**Mean ±SD**	**Mean ±SD**			
Gender (male/female)	17/30	13/30	1	0.207	0.649
Age (years)	26.12 ± 5.06	26.27 ± 5.69	88	0.140	0.889
Age range (years)	18–35	22–34	-	-	-
Education (years)	19.75 ± 4.18	20.30 ± 3.97	88		
Handedness (right/left)	47/0	43/0	-	-	-
HAMD	14.66 ± 5.60	2.37 ± 2.45	88	16.060	<0.001
HAMA	10.55 ± 5.18	0.767 ± 0.868	88	12.220	<0.001

*MMD, mild-to-moderate depression; HCs, healthy controls; HAMD, hamilton depression; HAMA, hamilton anxiety; WMS, wechsler memory scale*.

**Table 2:** Comparison of depression variables between patients with MMD after 8 weeks SG-treatment and HCs after 8 weeks (mean ± SD).

	**MMD patients**				**HCs**			
	**Baseline**	**8-weeks**	**t**	***p***	**Baseline**	**8-weeks**	**t**	***p***
HAMD	14.66 ± 5.60	6.31 ± 3.72	7.648	<0.001	2.37 ± 2.43	1.79 ± 1.99	1.211	0.199
HAMA	10.55 ± 5.18	4.62 ± 2.97	6.065	<0.001	1.97 ± 2.01	1.48 ± 1.71	1.218	0.298

*MMD, mild-to-moderate depression; HCs, healthy controls; HAMD, hamilton depression; HAMA, hamilton anxiety*.

### Tract-based spatial statistics

**Figure 2:**
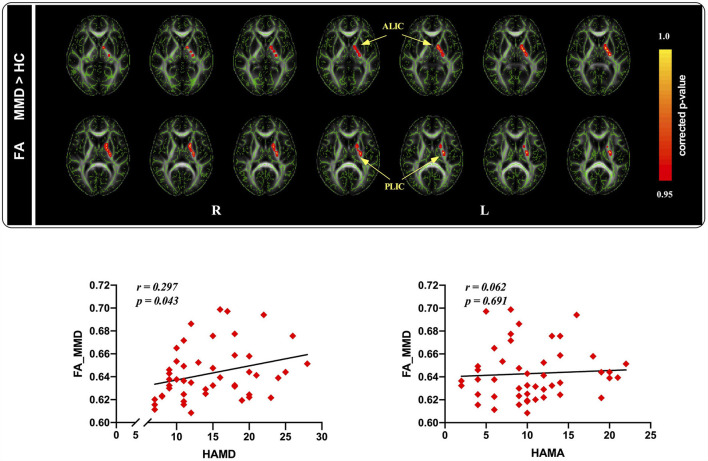
Tract-based spatial statistics maps are shown the FA differences between the two groups and the correlation between FA and HAMD as well as HAMA ratings in MMD patients. (Top): Red represents regions with significantly increased FA in MMD patients, compared with HCs. (Bottom): FA values were significantly correlated with HAMD ratings, whereas not related to HAMA ratings in MDD patients. FA, fractional anisotropy; HAMD, hamilton depression; HAMA, hamilton anxiety; MMD, mild-to-moderate depression; HCs, healthy controls.

At the baseline, FA values in patients with MMD were significantly increased in the regions of the anterior limb of the internal capsule (ALIC) and posterior limb of the internal capsule (PLIC) compared to those in HCs (Figure 2).

After 8 weeks of SG treatment, FA values were significantly decreased in the regions of the ALIC (MNI coordinates: Peak_(x/*y*/*z*)_ = 102, 126, 77; *t* = 3.720, *p* < 0.001; Table 3) and PLIC (MNI coordinates: Peak_(x/*y*/*z*)_ = 109, 117, 87; *t* = 18.880, *p* < 0.001; Table 3) compared to those at baseline. TBSS revealed no significant differences in FA values between patients with MMD and HCs.

**Table 3:** Main regions showing FA alterations in patients with MMD (clusters >50 voxels).

**MNI coordinates**	**Side**	**Voxels**	**White matter tract**	**FA mean (SD)**		**t-value**	***p*-value**
**Peak x/y/z**				**Baseline**	**8 weeks**		
• 102 126 77	• L	• 960	• ALIC	• 0.621 (0.043)	• 0.588 (0.043)	• 3.720	• <0.001
• 109 117 87	• L	• 523	• PLIC	• 0.694 (0.042)	• 0.558 (0.026)	• 18.880	• <0.001

*ALIC, anterior limb of the internal capsule; PLIC, posterior limb of the internal capsule; L, left; MNI, montreal neurological institute*.

To further understand the association between FA values and clinical variables in patients with MMD, we conducted correlation analyses between FA values and depression and anxiety scores in the patient group. The results indicated that FA values were significantly positively correlated with HAMD scores (*r* = 0.297, *p* = 0.043; Figure 2), but not with HAMA scores (*r* = 0.062, *p* = 0.691; Figure 2) in patients with MMD.

### Gray matter volume

**Figure 3:**
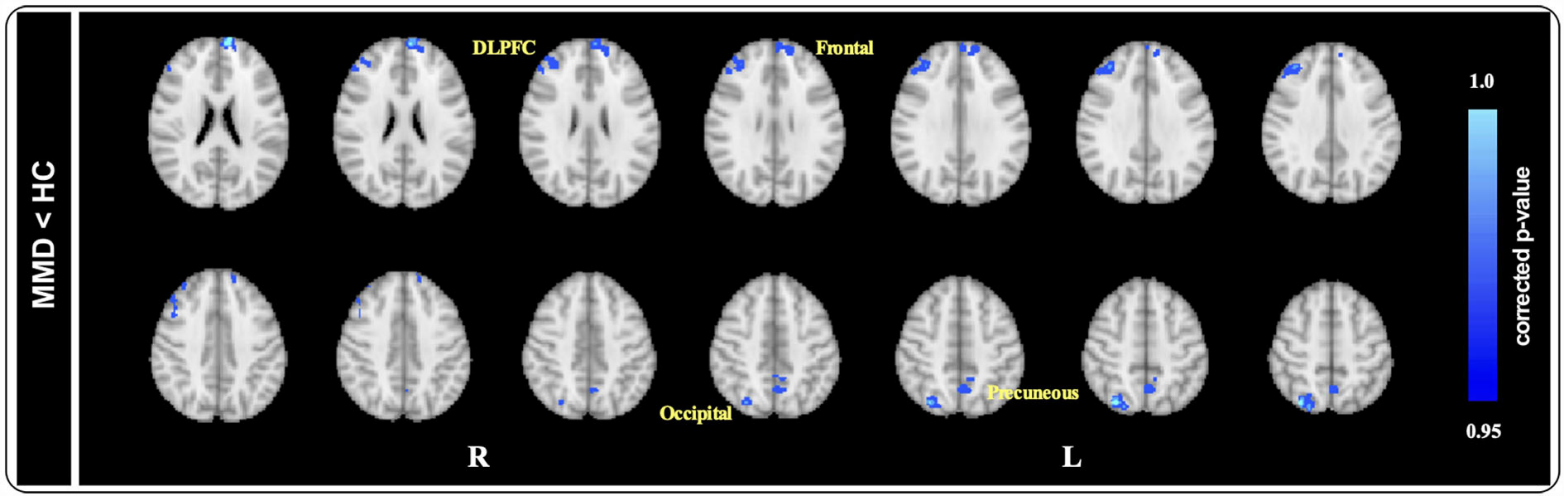
Voxel-based morphology comparison between MMD and HCs. After controlling the effects of age and gender by adding them as covariates, ACOVA showed the gray matter volume of the DLPFC, frontal cortex, occipital cortex, and precuneus were significantly smaller in MMD patients than in HCs (*p* < 0.05, TFCE corrected). DLPFC, dorsolateral prefrontal cortex; HAMD, hamilton depression; MMD, mild-to-moderate depression; HCs, healthy controls.

At baseline, after controlling for the effects of age, sex, and total brain size, we observed that the GM volume of the dorsolateral prefrontal cortex (DLPFC), frontal cortex, occipital cortex, and precuneus was significantly smaller in patients with MMD than in HCs [*p* < 0.05, threshold-free cluster enhancement (TFCE) corrected; Figure 3].

After 8 weeks of SG treatment, GM volume was significantly increased in the regions of the Frontal gyrus (MNI coordinates: Peak_(x/y/z)_ = 50, 94, 43; *t* = 4.402, *p* < 0.001; Table 4), DLPFC (MNI coordinates: Peak_(x/y/z)_ = 28, 84, 50; *t* = 7.416, *p* < 0.001; Table 4), precuneus (MNI coordinates: Peak_(x/y/z)_ = 46, 33, 57; *t* = 5.766, *p* < 0.001; Table 4), and Frontal gyrus (MNI coordinates: Peak_(x/y/z)_ = 29,83,26; *t* = 12.650, *p* < 0.001; Table 4) compared to those at baseline. There were no significant differences in GM volume between patients with MMD and HCs.

**Table 4:** Main regions showing gray matter volumes alterations in patients with MMD (clusters >50 voxels).

**Area**		**MNI coordinates**	**Voxels**	**GMV mean (SD)**		**t-value**	***p*-value**
**Area**	**Side**	**Peak x/y/z**		**Baseline**	**8 weeks**		
Frontal gyrus	R	50 94 43	488	0.401 (0.068)	0.489 (0.119)	4.402	<0.001
DLPFC	R	28 84 50	423	0.426 (0.065)	0.591 (0.138)	7.416	<0.001
Occipital cortex	R	30 27 60	369	0.451 (0.101)	0.491 (0.078)	2.149	0.034
Precuneus	L	46 33 57	218	0.543 (0.096)	0.669 (0.115)	5.766	<0.001
Frontal gyrus	R	29 83 26	186	0.192 (0.031)	0.335 (0.071)	12.650	<0.001

*DLPFC, dorsolateral prefrontal cortex; GMV, gray matter volume; L, left; R, right; MNI, montreal neurological institute*.

**Figure 4:**
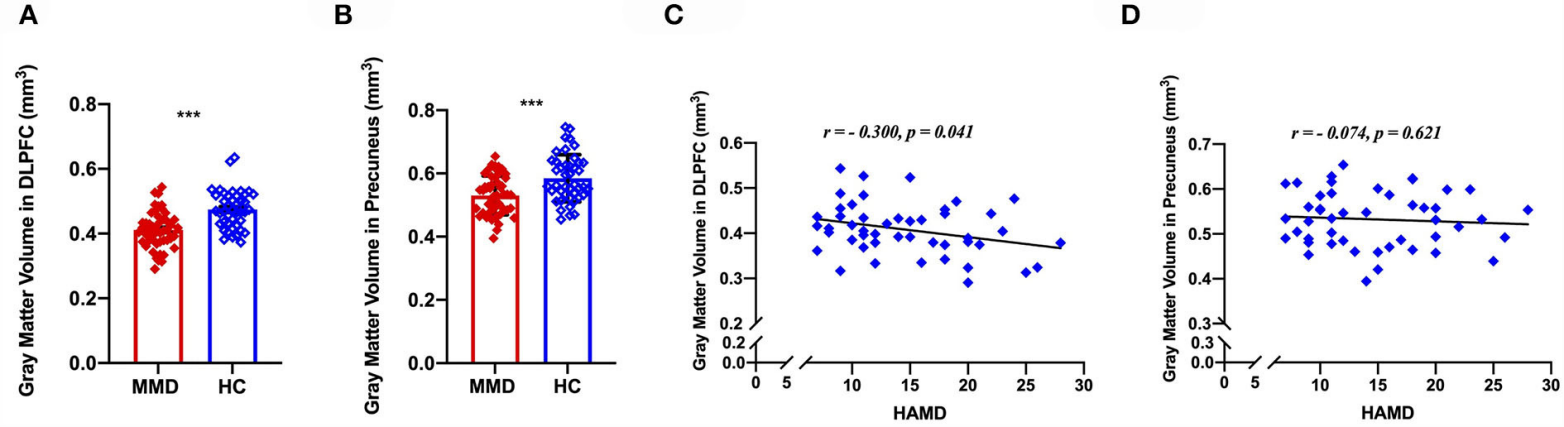
Voxel-based morphology comparison between MMD patients and HCs, and correlations between the reduction volumes and depression symptoms. The gray matter volume of DLPFC was significantly smaller in MMD patients than in HCs **(A)**, meanwhile, the gray matter volume of precuneus was significantly smaller in MMD patients than in HCs **(B)**. Scatter plots indicating that the gray matter volume of DLPFC was negatively correlated with HAMD scores in MMD patients **(C)**, whereas the gray matter volume of precuneus was not correlated with HAMD scores in MMD patients **(D)**. DLPFC, dorsolateral prefrontal cortex; MMD, mild-to-moderate depression; HCs, healthy control. ***, *p* < 0.001.

To understand the effects of depression on structural GM volume, correlation analyses were conducted to determine the relationship between GM volume abnormalities and depression scores. We extracted GM volume in the DLPFC and precuneus, correlation analyses indicating that the GM volume of DLPFC was negatively correlated with HAMD scores (*r* = 0.300, *p* = 0.041, Figure 4), whereas the GM volume of precuneus was not correlated with HAMD scores (*r* = 0.074, *p* = 0.621; Figure 4) in patients with MMD.

### Comparison of mild depression and moderate depression

It is worth noting that researchers have previously found that MDD patients with different severity have different neuroimaging changes in the default mode network (DMN) and the execution control network (ECN) (He et al., 2022). Thus, we divided patients with MMD into mild (*N* = 24) and moderate (*N* = 23) depression groups, and compared the GM volume and FA values of the two groups at baseline and 8 weeks of SG-treatment, respectively. At the whole brain level, there was no significant difference in structural characteristics between the two groups. In addition, we compared whether there were differences between mild and moderate depression groups in brain regions (FA: ALIC and PLIC; GMV: DLPFC, frontal cortex, occipital cortex, and precuneus) with structural differences between MMD and HCs. We found that there was no significant difference in those brain regions characteristics between the two groups at baseline (FA: *t* = 1.601, *p* > 0.05; DLPFC: *t* = 1.582, *p* > 0.05; frontal cortex: *t* = 1.032, *p* > 0.05; occipital cortex: *t* = 1.374, *p* > 0.05; precuneus: *t* = 0.403, *p* > 0.05) and 8 weeks of SG-treatment (FA: *t* = 0.442, *p* > 0.05; DLPFC: *t* = 0.997, *p* > 0.05; frontal cortex: *t* = 0.785, *p* > 0.05; occipital cortex: *t* = 0.893, *p* > 0.05; precuneus: *t* = 0.305, *p* > 0.05).

### Correlation between clinical symptoms and FA value alterations, as well as GM volume alterations

**Figure 5:**
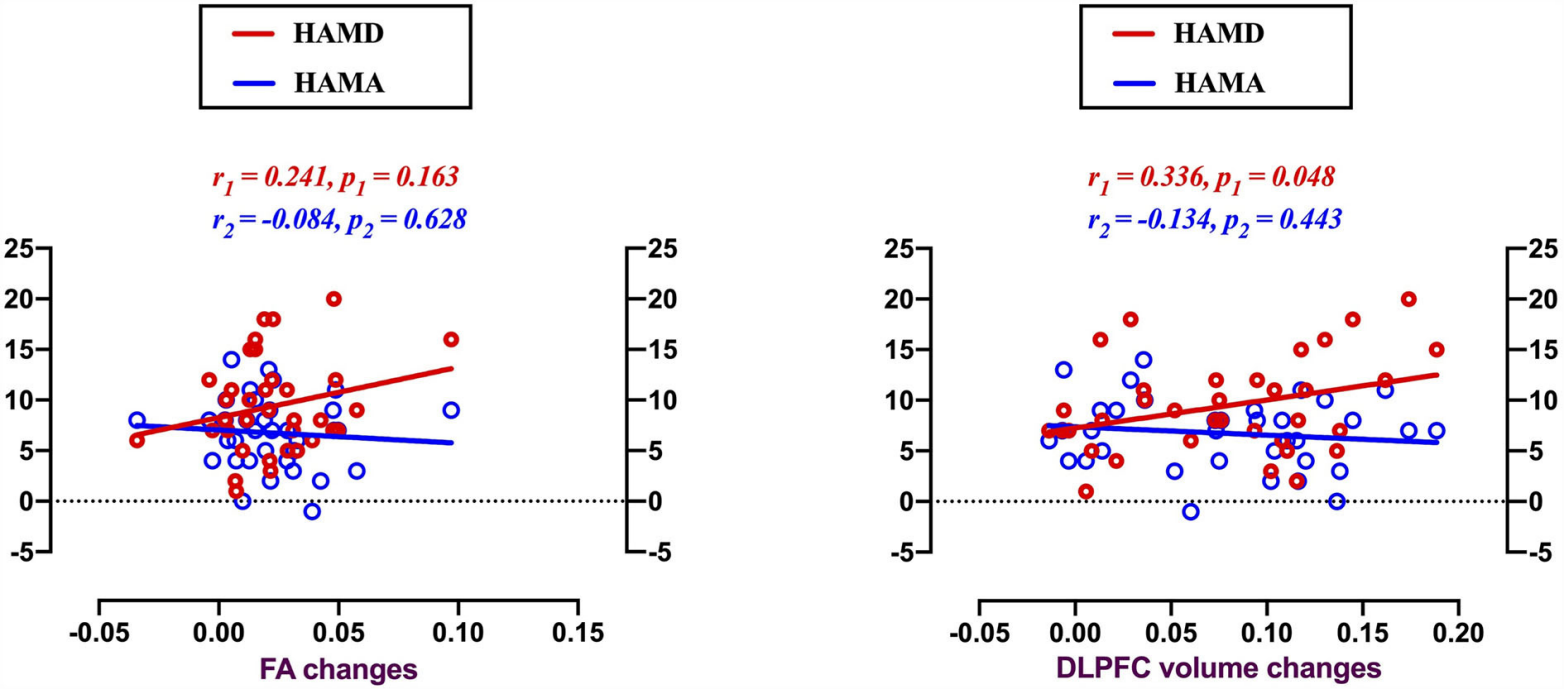
Voxel-based morphology comparison between MMD patients and HCs, and correlations between the reduction volumes and depression symptoms. Scatter plots indicating gray matter volume alterations in the DLPFC were found positively correlated with the HAMD score changes in MMD patients after 8 weeks SG-treatment (r = 0.336, *p* = 0.048). There were no significantly correlations between clinical symptom changes and FA value alterations.

Alterations of GM volume in the DLPFC was found to be positively correlated with HAMD score changes in patients with MMD after 8 weeks of SG treatment (Figure 5; *r* = 0.335, *p* = 0.048). And GMV in other three brain regions was not related to the HAMD scores changes: frontal cortex (*r* = 0.127, *p* = 0.942); occipital cortex (*r* = 0.250, *p* = 0.147); precuneus (*r* = 0.275, *p* = 0.109).

There were no significant correlations between clinical symptoms and FA value alterations.

## Discussion

The initial stage of a depressive disorder is MMD, and it is more common than severe depression in the general medical environment. The underlying mechanisms of MMD, especially the characteristics of brain structure, are poorly understood. In this study, we investigated brain structural alterations in patients with MMD compared with age- and sex-matched HCs and assessed the relationship between these brain structural alterations and clinical symptoms in patients with MMD. Our findings can be summarized as follows: at baseline, (1) FA values in patients with MMD were found to be significantly increased in the ALIC and PLIC, and FA values were significantly positively correlated with HAMD scores in patients with MMD; (2) patients with MMD showed smaller GM volume of the DLPFC, frontal cortex, occipital cortex, and precuneus than HCs, and the GM volume of the DLPFC was negatively correlated with HAMD scores. After SG treatment, we found that: (1) the HAMD scores decreased; (2) FA values were significantly decreased in the regions of the ALIC and PLIC compared to those at baseline and TBSS revealed no significant differences in FA values between patients with MMD and HCs; (3) the structural characteristics of the DLPFC in patients with MMD obtained at the 8th week were improved; for example, no significant differences were observed in GM volume of the DLPFC between the two groups. Our results provide neuroimaging evidence showing that SG is an effective treatment for patients with MMD. Moreover, alterations in GM volume after 8 weeks of SG treatment indicated a potential modulation mechanism in brain structural plasticity within the DLPFC in patients with MMD.

### Deficits of white matter microstructure and its relationship with clinical characteristics in MMD

Structural properties were obtained using voxel-wise TBSS. The most commonly used parameter in DTI research is FA, which reflects the structural integrity and geometry of axonal fibers (Gulani and Sundgren, 2006). Greater FA may reflect greater myelination of WM fibers, an increased number of myelinated fibers, smaller axonal diameter, or reduced neural branches within the MRI voxel (Boero et al., 1999; Beaulieu, 2002). In this study, the pattern in patients with MMD was different from that in HCs; increased FA values were observed in the ALIC and PLIC in MMD.

The ALIC in human and non-human primates carries ascending and descending fibers from the OFC and ACC. The OFC and ACC are associated with several psychiatric disorders, including obsessive-compulsive disorder, MDD, posttraumatic stress disorder, and addiction (Mayberg et al., 1997; Volkow et al., 2011; Milad and Quirk, 2012; Haber and Heilbronner, 2013). Importantly, diseases linked to the OFC and ACC show abnormal volume, FA, and diffusivity in the internal capsule, and some studies have demonstrated these abnormalities, specifically in the ALIC (Lang et al., 2006; Wobrock et al., 2008; Zou et al., 2008; de Souza Duran et al., 2009; Jia et al., 2010; Togao et al., 2010; Upadhyay et al., 2010; Nakamae et al., 2011; Zhu et al., 2011; Koch et al., 2014; Kuan et al., 2015). In addition, the ALIC is a WM bundle between the head of the caudate nucleus and the lenticular nucleus, which plays an important role in motivation, decision-making, and evaluation of the saliency of emotional and reward stimuli (Henderson et al., 2013). Consistent with our findings, Hyett et al. found that FA was distinctly increased in the right anterior internal capsule in patients with melancholia compared to controls (Hyett et al., 2018), and Coloigner et al. reported patterns of increased FA in the splenium of the corpus callosum and PLIC in patients with depression (Coloigner et al., 2019). In addition, researchers also found consistent results in other mental diseases, such as schizophrenia, obsessive-compulsive disorder, and bipolar depression; that is, the FA values of ALIC and PLIC in patients were increased compared with those in HCs (Versace et al., 2010; Lochner et al., 2012; Alba-Ferrara and de Erausquin, 2013). In patients with depression, a higher FA in the ALIC and PLIC was associated with more severe overall clinical symptoms, which is consistent with previous studies that have shown a strong association between WM disruptions in these regions and the symptoms of depression. Since FA values were significantly positively correlated with HAMD scores in patients with MMD after 8 weeks of SG treatment, TBSS revealed that there were no significant differences in FA values between patients with MMD and HCs. Similarly, behavioral data showed no significant difference in HAMD scores between patients with MMD and HCs. The Chinese herbal medicine SG is mainly composed of *Acanthopanax* and *H. perforatum*. *Acanthopanax*, commonly known as Ciwujia or Siberian ginseng, is a traditional Chinese medicine that ameliorates cognitive dysfunction induced by cholinergic blockade (Li et al., 2016). *H. perforatum*, also known as St. John's wort, has been used as an antidepressant agent and cognitive enhancer in rodents (Avila et al., 2018). *H. perforatum* has been shown to induce positive effects on both mood and short-term verbal memory (Yechiam et al., 2019). The main drug components of SG have good therapeutic effects on the clinical symptoms of MMD. Therefore, after 8 weeks of SG treatment, the clinical symptoms of patients with MMD were reduced, and there was no significant difference compared with HCs, while the FA positively correlated with clinical symptoms was also reduced, and there was no significant difference compared with HCs.

### Deficits of GM volume and its relationship with clinical characteristics in MMD

After taking sex, age, and baseline volume as covariates, brain GM volume of the DLPFC, frontal cortex, occipital cortex, and precuneus were significantly smaller in patients with MMD than in HCs. Consistent with our findings, several previous studies have reported differences in GM volume between patients with depression and HCs. For example, Lyoo et al. found evidence of decreased GM volume in the anterior cingulate gyrus, left medial frontal gyrus, and right precentral gyrus (Lyoo et al., 2004). Most studies have identified decreased GM volume in a wide range of brain regions, including the pre-supplementary motor area, parietal-temporal regions, frontal cortex, temporal cortex, cingulate cortex, insular cortex, parahippocampal gyrus, hippocampus, cerebellum, and OFC (Tang et al., 2007; Cheng et al., 2010; Lai et al., 2010; Zou et al., 2010; Peng et al., 2011; Ma et al., 2012; Wang et al., 2012; Zhang X. et al., 2012; Kong et al., 2014). Neuroimaging studies have produced interesting findings on the structural effects of antidepressant treatment in the prefrontal areas. The medial frontal gyrus and DLPFC volumes predict remission after antidepressant treatment (Chen et al., 2007; Yucel et al., 2009; Lorenzetti et al., 2010). Furthermore, effective treatment with fluoxetine and sertraline results in enlargement of the middle frontal gyrus, DLPFC, and OFC (Smith et al., 2013; Kong et al., 2014). Accordingly, remission has been shown to correlate with more preserved volumes of the anterior cingulate gyrus, dorsomedial prefrontal gyrus, and DLPFC over time (Frodl et al., 2008; Gunning et al., 2009). Furthermore, correlation analyses indicated that the GM volume of the DLPFC was negatively correlated with HAMD scores. Depression is characterized by dysfunction in cognitive, emotional, and behavioral processes related to emotion processing and regulation (Joormann and Gotlib, 2010). Thus, the study of neurobiologically informed models of depression focuses on brain regions underlying normative functioning of these processes (Drevets et al., 2008), including examining structural differences in these regions between patients with depression and healthy. The DLPFC is a large and functionally heterogeneous brain region (Glasser et al., 2016) and is involved in emotional and cognitive processing, and is one of the key players in the pathophysiology of mood disorders (Matsuo et al., 2019). Meta-analyses of MR images from patients with MDD have shown that compared with healthy subjects, patients with MDD have decreased GM volumes in the right DLPFC (Bora et al., 2012). In one another previous meta-analysis study, patients with MDD were shown to have reduced activation in the DLPFC (Fitzgerald et al., 2008). Besides, excitatory repetitive transcranial magnetic stimulation (rTMS) over the left DLPFC (Gu and Chang, 2017) or anodal left/cathodal right DLPFC transcranial direct current stimulation (tDCS) (Valiengo et al., 2017) have been shown to reduce depressive symptoms in post-stroke depression patients. This provides evidence for the negative correlation between DLPFC and MDD symptoms from the perspective of non-pharmacological interventions. In fact, stimulation at the DLPFC is the FDA-approved antidepressant intervention (Lan et al., 2016), and is based on the observation that, in depression, this structure has impaired functional connectivity (Sheline et al., 2010; Liston et al., 2014) and that left prefrontal cortical strokes increase the risk of depression (Shimoda and Robinson, 1999). The above studies are in line with a common idea that the structural changes of DLPFC either predict the onset of depression or reflect the damage caused by repeated emotions in depression patients.

### A potential modulation mechanism in brain structural plasticity within the DLPFC in MMD

Multiple linear regression analysis showed that, as a typical mental disorder, MMD also has serious brain structural deficits, such as reduced GM volume, in addition to its etiology. Previous studies on depression have reported consistent findings regarding the association between the left DLPFC and clinical outcomes. Several lines of evidence suggest a role of the DLPFC in the phenotypes, onset, duration, and treatment efficacy of depression (Kinou et al., 2013; Tsujii et al., 2016). For example, previous studies have shown that patients with MDD have a significantly decreased GM volume in the left DLPFC, which plays a key role in positive emotional judgment. The significantly decreased GM volume we observed in the DLPFC of depression was in agreement with the clinical symptoms of diminished interest or pleasure in almost all activities in depression (Qi et al., 2022), supporting the view that DLPFC plays a crucial role in the symptomatology of depression (Koenigs and Grafman, 2009). This view is further supported by functional MRI study, which indicates that DLPFC activity was decreased and the functional connection (FC) between the amygdala and the DLPFC was reduced in untreated depression patients performing cognitive function tests (Siegle et al., 2007), and that in depression patients there was a widespread reduction in FC between the left DLPFC and other brain areas (Liston et al., 2014). Our results also provide evidence that DLPFC is a key brain region in the pathogenesis and rehabilitation of depression. At baseline, after controlling for the effects of age, sex, and total brain size, we observed that the GMV of the DLPFC, frontal cortex, occipital cortex, and precuneus was significantly smaller in patients with MMD than in HCs. And there were no significant differences in GMV between patients with MMD and HCs after 8 weeks of SG treatment. This means that after treatment, the structure of the above four brain regions has changed, that is, GMV has been improved. Then, we made a correlation analysis between the alterations of GMV in these four brain regions and the HAMD score changes. The results showed that only the alteration of GMV in DLPFC was positively correlated with the HAMD score change. We consider that the possible reason for this result is that as one of the key brain regions in the pathogenesis and rehabilitation of depression, DLPFC is a key hub involved in cognitive control, working memory, and emotion regulation. This means that DLPFC may be more sensitive to SG treatment than the other three brain regions. And in line with previous reports, alterations in GM volume after 8 weeks of SG treatment indicated a potential modulation mechanism in brain structural plasticity within the DLPFC in patients with MMD. In addition, PFC abnormalities observed in patients with MDD or rodent models are partly corrected by antidepressants. An 8-week escitalopram treatment indicated reduced irregular high functional connectivity in the bilateral dorsal medial PFC in patients with MDD, and a 2-week administration of a selective serotonin reuptake inhibitor, fluvoxamine, restored dendritic length, and spine densities, but not cortical thickness after early life stress exposure (Lyttle et al., 2015; Wang et al., 2015). Overall, our data suggest that the degree of depression in MMD largely depends on the GM volume of the DLPFC in a given patient.

### Limitations

First, the sample size of patients was limited and involved patients with varying severity and duration of disease, which limits the investigation of the detailed role of each mechanism in the development of MMD. Second, the causal relationship between pathophysiological factors and depression severity should be detected not only based on a large sample of patients but also using some effective experimental designs, for example, a longitudinal study to monitor the transition from MMD to major depression. Of course, such a study would be fraught with additional challenges based on time effects and the likely contaminating effects of medication or therapeutic interventions to address depression. Third, in addition to the severity of depression, other factors such as the duration of depression and the number of depressive episodes can regulate the brain structure. Unfortunately, we have not collected information on these factors. Finally, our study may have underestimated the extent of structural changes associated with depression. Insufficient sensitivity may have arisen because of the limited sample size. Thus, if there were mood-state-dependent changes in GM volume, we may not have detected them because of insufficient power.

### Further prospects

Despite these limitations, this study provides further evidence that the PFC might play a key role in the regulation of depression in the pathophysiology of MMD. Future studies using a larger sample size or longitudinal studies are needed to replicate and generalize these results across patients with severe depression. More detailed insights into the structural and functional brain plasticity underlying MMD will shed light on the development of new and more targeted treatment options in clinical practice.

## Data availability statement

The raw data supporting the conclusions of this article will be made available by the authors, without undue reservation.

## Ethics statement

The studies involving human participants were reviewed and approved by the Ethics Committee of Shanxi Medical University. The patients/participants provided their written informed consent to participate in this study.

## Author contributions

YL: data curation, formal analysis, and writing—original draft. JW: data curation and writing, reviewing, and editing the manuscript. XY: investigation and resources. HL: supervision and writing, reviewing, and editing the manuscript. All authors contributed to and approved the final manuscript.

## Funding

This work was supported by the National Key Research and Development Program of China (2016YFC1307004), the National Natural Science Foundation of China (81971601, 82101610, and 81571319), the Wu Jieping Foundation (320.6750.18283), the Special Project for Guiding the Transformation of Scientific and Technological Achievements in Shanxi Province (201904D131020), and the Multidisciplinary Team for Cognitive Impairment of Shanxi Science and Technology Innovation Training Team (201705D131027).

## Conflict of interest

The authors declare that the research was conducted in the absence of any commercial or financial relationships that could be construed as a potential conflict of interest.

## Publisher's note

All claims expressed in this article are solely those of the authors and do not necessarily represent those of their affiliated organizations, or those of the publisher, the editors and the reviewers. Any product that may be evaluated in this article, or claim that may be made by its manufacturer, is not guaranteed or endorsed by the publisher.
